# Surgical treatment of renal cell carcinoma recurrence at the renal fossa following radical nephrectomy

**DOI:** 10.1590/S1516-31802008000300011

**Published:** 2008-05-01

**Authors:** Carlos Márcio Nóbrega de Jesus, Filemón Anastásio Silva Casafus, Aparecido Donizetti Agostinho

**Keywords:** Kidney neoplasms, Neoplasm recurrence, local, Retroperitoneal space, Surgery, Recurrence, Neoplasias renais, Recidiva local de neoplasia, Espaço retroperitoneal, Cirurgia, Recorrência

## Abstract

**CONTEXT:**

Isolated renal cell carcinoma recurrence at the renal fossa is a rare event. This condition occurs in 1 to 2% of radical nephrectomy cases. It is usually seen in postoperative follow-up imaging examinations such as abdominal computed tomography or abdominal ultrasound. There is controversy among urologists and oncologists regarding the best way to treat this rare situation, because of the few cases in the literature.

**CASE REPORT:**

We report on a case of isolated recurrence at the renal fossa due to renal cell carcinoma (RCC), four and a half years after radical nephrectomy, without evidence of metastases in other organs. The diagnosis was made from abdominal tomography performed during outpatient follow-up, in which a retroperitoneal mass was observed in the renal fossa. Excision was carried out by means of a subcostal transversal incision, without complications. One and a half years after the procedure, there was evidence of metastasis in the left lung and, six months later, another recurrence at the ninth anterior right rib, while the patient remained asymptomatic. Aggressive surgical treatment is a good method for controlling this rare situation of single retroperitoneal RCC recurrence. Abdominal tomography must continue to be performed over long periods of follow-up, to monitor for RCC following radical nephrectomy, in order to diagnose any late retroperitoneal recurrences. These must be treated as single RCC metastases.

## RESUMO

**CONTEXTO:**

A recorrência local única do carcinoma renal de células claras em seu leito renal após nefrectomia radical é um evento raro. Estima-se que essa situação ocorra em 0,8% a 3,6% do total de procedimentos. Comumente, seu diagnóstico é realizado através de tomografia computadorizada de abdômen ou ultra-som renal usados no acompanhamento desses pacientes. É polêmico qual o melhor tratamento dessa rara condição entre urologistas e oncologistas devido aos poucos relatos em literatura.

**RELATO DE CASO:**

Relatamos um caso de recidiva neoplásica única no leito renal após quatro anos e meio da nefrectomia radical por adenocarcinoma de células claras, sem evidência de metástases a distância em outros órgãos. O diagnóstico foi realizado por meio de tomografia abdominal em acompanhamento ambulatorial, observando-se massa retroperitoneal em topografia renal. A massa foi retirada por meio de uma incisão subcostal ampliada, em cirurgia sem intercorrências. O paciente evoluiu bem no pós-operatório. Após um ano e meio do procedimento, foi evidenciada uma metástase no pulmão esquerdo, e seis meses após, outra recorrência metastática na nona costela anterior à direita, mesmo com paciente totalmente assintomático. O tratamento cirúrgico agressivo em recorrência local única é um bom método para controlar essa rara doença. Tomografia computadorizada de abdômen deve ser feita em acompanhamento de carcinoma renal por longos períodos após a nefrectomia radical para o diagnóstico de recorrências tardias e o tratamento deve ser feito como o de uma metástase recorrente única.

## INTRODUCTION

The behavior of renal cell carcinoma (RCC) following radical nephrectomy is sometimes unforeseeable. Recurrences usually occur within the first two years after the surgery and they are more common in bones and in lungs, among other organs.^1^ Local single recurrence following radical nephrectomy is a rare event and occurs in 0.8 to 3.6% of such cases.^2,3^

Because RCC is a rare condition without a standardized treatment and chemotherapy and radiotherapy produce poor results, there is interest in the management of this small select group. In this light, we report a case of solitary RCC recurrence following radical nephrectomy that was treated by excision with a good outcome despite other, subsequent recurrences during the follow-up.

## CASE REPORT

**Figure 1:**
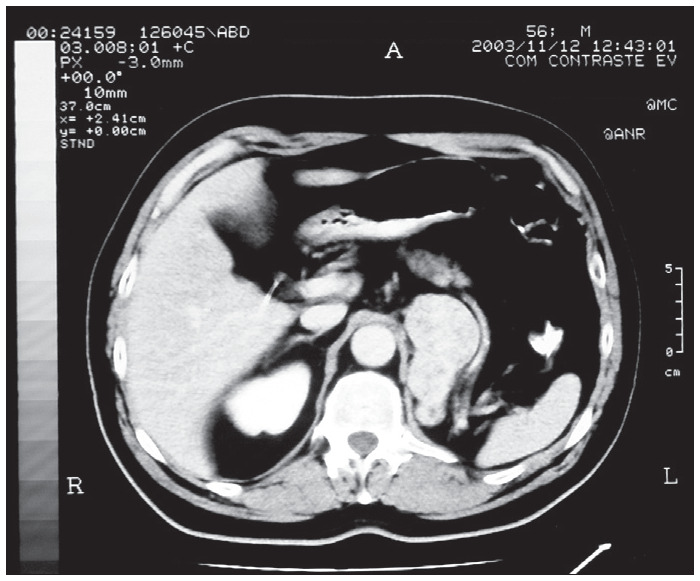
Abdominal tomography using endovenous infusion of contrast into left renal topography showing solid recurrence of renal cell carcinoma. The diagnosis was made four and a half years after left radical nephrectomy.

An asymptomatic 57-year-old white man had been undergoing follow-up at our institution for two years following left radical nephrectomy due to RCC grade 3 that had been performed at another service. After four and a half years of follow-up, a left retroperitoneal mass measuring 7 cm was observed by abdominal computed tomography (CT) (Figure 1). In previous imaging examinations, there had not been any evidence of neoplasm recurrences.

**Figure 2:**
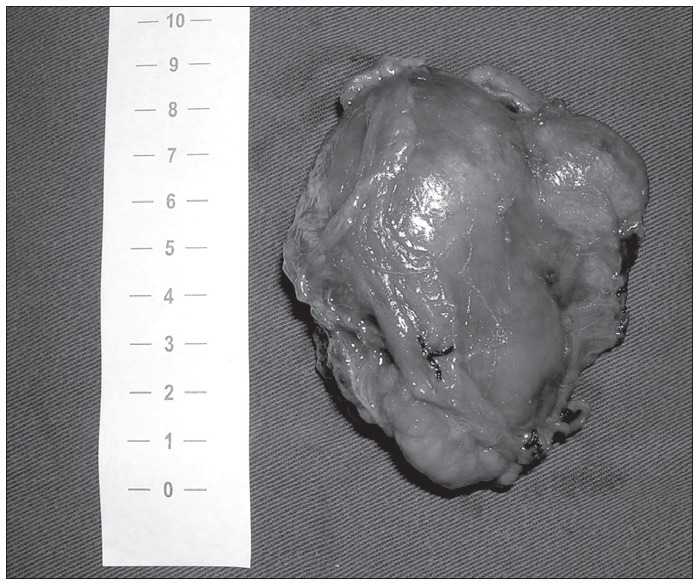
Macroscopic appearance of the specimen in Figure 1.

The possibility of metastases in other organs was ruled out by means of chest X-ray and bone scintigraphy. Surgical exploration was scheduled with the patient’s consent and his recognition of the risks and the possibilities of recurrence following the surgery. A left subcostal incision was performed and a 9 cm solid mass adhering to the aorta was observed. It was removed with minimal blood loss (Figure 2). The patient recovered well and was discharged on the fourth day.

Histological analysis confirmed that this was a case of RCC grade 3. Abdominal and chest CT examinations were performed six, twelve and eighteen months later, and no recurrence at the renal fossa was observed. However, at the eighteen-month follow-up, a 3 cm rounded nodule with irregular borders was seen in the upper part of the left lung on chest CT. This one was removed by means of left lung lobectomy a few days later. The diagnosis of RCC metastasis was confirmed. Six months later, a bone scan showed a lesion at the ninth anterior right rib. Since then, the patient has remained totally asymptomatic. He has been treated with endovenous bisphosphonates, with a good response so far.

## DISCUSSION

Isolated RCC retroperitoneal recurrence following radical nephrectomy is a rare event. In a retrospective study performed by Itano et al.,^2^ 30 patients had single local RCC recurrence, corresponding to 1.8% of the cases of radical nephrectomy performed at that institution. In their study, although the survival rate was low (28% reached five years), the patients who underwent surgery presented an improvement in survival rate of 51%, compared with 13% among those who were only observed.^2^

In another study, Schrodter et al. presented 16 patients with isolated retroperitoneal recurrence that was diagnosed by ultrasound or CT. All of them were treated by a surgical approach.^3^ Three of them did not have any neoplasm recurrence and they were considered to be false positives. In two operated cases, accessory spleen was found and, in the third, scar tissue granuloma was found around the vascular ligatures of the former renal hilus, as a neoplasm recurrence.

Thirteen of Schrödter’s cases were followed up: seven died and six remained alive. The mean time until recurrence was 45 months (range: seven to 224). Among the patients who were still alive, the time until recurrence following nephrectomy was significantly longer and size of the recurrence was smaller. The authors concluded that, although most of these patients would occasionally have and might die of metastatic disease, an aggressive surgical approach was justified.^3^

Because RCC is a type of neoplasia with little response to adjuvant treatments, surgery must be the first choice in a case of single retroperitoneal recurrence following radical nephrectomy, in the absence of metastatic disease in other places. Immunotherapy did not improved the survival among the select group of patients described above.^2,3^

In the present case report, we used the open transperitoneal route because we believed that this was the best approach for reaching and removing the mass, since it was in close contact with the aorta. However, a hand-assisted approach has already been successfully performed to remove a recurrent tumor following radical nephrectomy, thereby offering the patient a minimally invasive procedure.^4^ Although we have performed this technique in our institution, we believe that the open approach facilitated safe exposure and release of the tumor in the present case.

Routine use of abdominal CT is justified, even a long time after radical nephrectomy, because of the possibility of late recurrence.^5-7^ In our institution, we have been using abdominal CT every six months over the first two years of follow-up, and yearly thereafter. Chest CT has not been used routinely. It has just been used in cases of doubtful chest X-ray. We recommend physical examination, biochemical tests, chest X-ray plus abdominal CT.

Despite the lack of randomized studies to prove which type of therapy is best for this kind of patient, surgery seems to be the best way to treat solitary local recurrence at the renal fossa following radical nephrectomy, particularly in patients presenting good health status, longer time until recurrence and smaller size.^3^ Other studies using new therapies with base controls are needed in order to establish what the most effective therapy would be.

## CONCLUSION

In summary, in cases in which local recurrence occurs in single form, surgical treatment must be offered to extend survival for this select group of patients and maybe provide a better life quality.
